# Making Home: The Role of Social Networks on Identity, Health, and Quality of Life Among Rural Lesbian and Gay Older Adults

**DOI:** 10.1093/geroni/igad082

**Published:** 2023-07-27

**Authors:** Marc Aaron Guest, Elizabeth G Hunter, Nancy E Schoenberg

**Affiliations:** Center for Innovation in Healthy and Resilient Aging, Arizona State University, Phoenix, Arizona, USA; College of Public Health, University of Kentucky, Lexington, Kentucky, USA; College of Medicine, University of Kentucky, Lexington, Kentucky, USA

**Keywords:** Egocentric data collection, Environmental gerontology, LGBTQ health, Social support, Rural aging

## Abstract

**Background and Objectives:**

Gay and lesbian older persons face a host of health inequalities related to their identity as they age. Challenges to health access and appropriate social support may be even more exacerbated for those living in rural environments; this may be due to the lack of supportive and affirming social connections. This project aimed to explore and describe the social networks and the relationship of these social networks to identity, health, and quality of life of gay and lesbian individuals in rural communities.

**Research Design and Methods:**

Social network data on network type, size, and social capital were collected and supplemented by quantitative questionnaires relating to health, quality of life, marginalization, and identity.

**Results:**

Participants (*N* = 25) were recruited from three states. Thirteen participants self-identified as gay and 12 as lesbian. All but one identified as non-Hispanic White. The average age of all participants was 60.32 years. Findings indicate that rural gay and lesbian individuals develop networks with little consideration for network members’ acceptance of their identity. Participants reported an average network size of 9.32 individuals. Gay men reported higher perceptual affinity (.69) than lesbian participants (.62). Lesbian networks showed significantly (*p* = .0262) greater demographic similarity (.58) than aging gay men’s networks (.55). Aging gay men (.89) reported statistically stronger (*p* = .0078) network ties than aging lesbian females (.78). Among participants in this study, network size is not correlated with the health and quality of life of rural aging lesbian and gay individuals. Still, personal identity congruence does appear to relate to health and quality of life.

**Discussion and Implications:**

The findings highlight the collective need to continue research into sexual minority aging and rural sexual minority aging, particularly employing novel methods.

**Translational Significance:** Our article explores an undeveloped area of research, how social networks can influence identity, health, and quality of life among older rural gay and lesbian populations. In doing so, we identify challenges faced by older rural gay and lesbian individuals in developing supportive networks, which are associated with better health and well-being. This work informs how providers and communities can create supportive systems for this population. Furthermore, it identifies additional research needs for this population.

In the United States, older adults living in rural environments experience more significant health challenges than those living in urban or suburban settings. Challenges faced by those living in rural environments include lack of access to health care facilities, higher poverty rates, lower rates of educational attainment, higher rates of chronic disease and cancers, and less dense social networks (1–4). These challenges result from a host of social drivers of health, including limited access to funding, lower socioeconomic career and opportunities, and increase across the lifespan (5). Marginalized groups, such as the gay (men attracted to the same sex) and lesbian (women attracted to the same sex) population, may face greater health inequality in rural areas than their urban counterparts as they seek to manage their identity as a sexual minority population (6,7). In the coming decades, more gay and lesbian individuals than ever before will enter old age (7,8). Research does indicate that gay and lesbian aging individuals face unique challenges related to health care access, service delivery, and social support due to a lifetime of encountering homophobia and discrimination associated with their sexual identity (9,10). However, there exists limited scholarship on these populations; in part, this is due to assumptions that this population does not exist difficulties in recruitment, and an emphasis on urban lesbian and gay populations.

Gay and lesbian individuals face a multitude of health inequalities (11–13). Overall, lesbian and gay individuals are at a higher risk of social isolation and have higher rates of disability, more psychological distress, weaker immune systems, lower than average incomes and standards of living, and fewer opportunities for advancement; also, they utilize fewer social services and face longer lifetime discrimination and victimization than nonlesbian, gay, bisexual, and transgender individuals (11,14,15). As a result, lesbian and gay individuals often face poorer health outcomes than their heterosexual counterparts (16,17). Lesbians have higher rates of disability, cardiovascular disease, obesity, and poorer general health than nonlesbian individuals (18–21). Gay men have a higher risk of cancer and HIV (11,16). These challenges, particularly related to isolation, may be exacerbated in rural communities due to a lack of available supportive individuals and resources. Aging gay and lesbians with racial/ethnic minority backgrounds face even more significant disparities (22,23). These disparities are amplified due to existing disparities between non-Hispanic White and minority communities (24,25). They are also exacerbated due to the exclusion of minority populations within the broader LGBTQ (lesbian, gay, bisexual, transgender, queer) community and often in rural communities (24,26,27).

Conceptualizing the challenges LGBTQ, and especially LGBTQ people of color, face requires understanding individuals in terms of their multiple competing identities and the potential health outcomes that result from these identities. Sexual identity is only one part of an individual’s overall identity (9,28). Individuals adopt a sexual identity (in the case of our study, gay or lesbian) through self-realization and actualization. People may internalize and externalize their sexual identity and thus disclose it to others, or they may internalize only their sexual identity and not disclose it to others (9). Disclosure decisions have implications beyond social connections and include the type and quality of health care individuals receive (29). Intersectionality seeks to explain how individuals make sense of their multiple identities and the associated benefits and oppression stemming from them (30). Intersectionality serves to understand how individuals identify themselves concerning others and the social contexts in which they adopt particular identities. Individuals who identify as LGBTQ must navigate a complex social web of identification and identity formation (6,31). Further, they must contend with other identities, such as ascribed identity or self-identification as a rural individual. There is a need to explore the aging process and age-related burdens experienced by aging rural gay and lesbian individuals to address the existing disparities. Multiple determinants of health inequality exist, including socioeconomic status, housing and environment, educational status, and social contexts and contacts. Understanding how individuals navigate these environments and their social networks’ effect on these determinants is an understudied area of scholarship. In this study, we examine the influence of social contexts and social contacts on health, identity, and quality of life using a network science approach. Network science provides an avenue for exploring and understanding the health of lesbian and gay individuals.

## A Primer on Social Networks

Social networks represent interactions and relationships among individuals that occur within a social environment or sociocultural context (32,33). Social networks consist of individuals (egos) and their contacts (those the ego interacts with). The strength and support provided will vary between the different contacts interacted with (34). Research has indicated that social networks have a powerful influence on health (35–37). Marginalized populations, such as lesbian and gay individuals, tend to have smaller, more homogenous (ie, consisting of similar individuals) networks that can limit the flow of information and resources (38). The development of social networks can influence and is influenced by the identity an individual develops (38).

Social networks link people to resources and knowledge and may increase social capital (39). Social capital, a social network of people and resources that provides support as individuals age (40,41), can be accrued as individuals develop networks throughout their life. Rural individuals may have fewer opportunities to establish multifaceted, diverse networks but may have more dispersed networks (42). Individuals aging in rural environments have fewer opportunities for developing social contacts (network contacts) than their urban counterparts (43). In part, this is due to the distance between individuals in rural areas, the potential for the lack of place to socialize, particularly LGBTQ-specific, and the aging of the rural populations. In the case of lesbian or gay individuals, the conflict between multifaceted networks composed of those who do and do not accept their sexual identity may result in individuals not recognizing gay or lesbian identity, due to a lack of exposure to other gay or lesbian individuals or fear of negative feedback. Individuals who do not disclose their gay or lesbian identity may have limited opportunities to locate affirming individuals or locations for fear of being identified or “outed” (having their identity disclosed without their permission). When internal and external identities are aligned, individuals with identity congruence can develop integrated and supportive social networks.

Lesbian and gay individuals report fewer social contacts than their heterosexual peers (38). The ability of gay and lesbian networks to provide resources and benefits may be reduced due to having fewer people in their networks; thus, less social capital or the unreliability of social relationships to accomplish a task (44). It has been demonstrated that as we age, our networks shrink (45). With fewer social contacts, rural aging gay and lesbian individuals may be even more negatively affected by shrinking networks over their life span, limiting their support in older age (46).

## The Purpose of This Project

This research was developed in response to the gaps in understanding the relationships among social networks, identity, health, and quality of life of gay and lesbian individuals, particularly in rural environments. Recognizing the need for additional work that examines and compares discrete sexual minority populations (ie, lesbian and gay) rather than the entirety of sexual and gender minority groups (ie, LGBTQ), we seek to provide information on 2 sexual minority groups (31,47). This study aimed to examine and describe the social network of aging lesbian and gay individuals living in rural communities and the relationship between social networks and identity, health, and quality of life. At the same time, we aimed to respond to the call for research that recognizes rural as worthy of study without comparison to urban environments (48). Little information exists about the health status of rural aging gay and lesbian individuals in the United States (19). Thus, the gained information can inform future research and interventions addressing the support of rural aging gay and lesbian individuals.

## Method

### Theoretical Foundations

A socioecological model of understanding health guides this research. This approach provides a guide for understanding an individual’s development and health through the broader societal and ecological systems in which they participate (49,50). The socioecological model emphasizes the individual’s interactions that support the development of social capital, including the cultural and political processes at play in affecting each person’s place within all aspects of society (51). This includes understanding an individual’s intersectional identities and how they allow for and deny access to specific resources, including health care and individuals. The socioecological model allows for examining how individuals influence others and are influenced by the systems surrounding them, including family, community, health, education, legal systems, and identities.

### Study Design

We employed a multimethod approach to data collection. Social network data on network type, size, and social capital were collected by interviews and supplemented by quantitative questionnaires relating to health, quality of life, marginalization, and identity, an approach common in egocentric network data collection (34,52). Participants were recruited from rural counties, as defined by the Rural–Urban Continuum Codes (RUCC), in Kentucky, West Virginia, and Tennessee (53). These states were selected; they were near the University of Kentucky and the authors could drive to the participants if needed. Rural residency was confirmed by home address. Participants were eligible if they self-identified as gay or lesbian, were age 50 and older, lived in one of the RUCC-identified counties, and expressed willingness to participate in the study. We selected a 50 and older age group to include in recognition of the multiple disadvantages experienced by this population throughout their lives, which often result in a shorter life span for LGBTQ individuals (54). Due to the region’s demographics, we expected most participants to be non-Hispanic White.

Recruitment occurred from September 2018 to February 2019. Although initial plans called for snowball sampling from an LGBTQ partner organization, this method had limited success as organizations had limited contact in rural communities. Social media advertisements, mainly through Facebook, resulted in the greatest participant recruitment. Fifteen of the 25 participants (60%) were recruited through social media advertisements on Facebook. The remainder were recruited through word of mouth and flyers. Participants received $30 for participating. Survey instruments were pilot tested with 4 aging (50+) lesbian and gay individuals. This study was approved by the University of Kentucky Institutional Review Board.

### Data Collection

Data collection took place during one in-person or over-the-telephone meeting based on the needs of the participants. Ten (*n* = 10; 40%) of the interviews took place in-person in the participant’s county of residence, with the remaining (*n* = 15; 60%) occurring by recorded telephone interviews. The same questionnaires were used in both instances with no revisions. Regardless of the place of data collection, the process remained the same. The interviewer went through the questions with the participant and marked their responses. The participant had a copy of the questions in front of them to follow along.

Initially, all data collection was to take place in-person using tablet-based data collection. The recruitment process made it clear that many participants felt uncomfortable meeting at a public location to discuss these sensitive issues (55). Nor did they feel comfortable inviting an unknown researcher into their home. As such, telephone interviews became one way to collect data and engage participants.

### Participant Demographic and Health Survey

During the meeting, participants completed a self-reported (survey) questionnaire that included demographics, health status, quality of life, rural identity, sexual orientation, identity congruence, and experiences of discrimination/homophobia.

Demographic data collected included age, sex, race/ethnicity, marital status, employment status, education, family size, and living situation. A modified version of the Centers for Disease Control (CDC) Behavioral Risk Factor Surveillance System (BRFSS) Survey standardized demographic questionnaire, with the 2-step method of collecting sexual and gender identity, was used for the demographic data collection (56–58). Health status was ascertained through the World Health Organization single-item, self-reported health question (59). The CDC BRFSS 2017 Disease-Specific questionnaire supplemented the WHO single-item self-reported health question in providing a broader picture of each participant’s health (57). The 2002 revised Flanagan Quality of Life Scale (QOLS) was used to examine and determine quality of life (60). We collected rural identity using the rural subscale of the Appalachian Identity Scale (61). This six-item tool assesses participant’s sense of belonging to rural areas with a higher score indicating greater rural identity. Identity congruence of sexual orientation and experiences of discrimination were assessed utilizing the Lesbian, Gay, Bisexual Identity Scale (LGBIS) Identity Appraisal (positive associations) and Identity Stigma (adverse associations) subscales (62), and the Nebraska Outness Scale (NOS) (63). The LGBIS subscales provided a measure of individual’s internal acceptance of their lesbian or gay identity. The NOS supplements the LGBIS by including specific measures of concealment and disclosure, adding a focused external dimension to identity congruence, with specific questions relating to level of disclosure across multiple groups (63). All measures were valid and reliable in their original format (42–48).

### Social Network Data Collection

Social network data were collected through the ENSO software package (64). The ENSO program was beta-tested in the field during this study. ENSO is a tablet-based network data collection instrument based on universal design principles. It automatically recalls participants’ connections entered on the first screen and allows the participant to drag and drop participants into categories. Other data can be collected through scale, dropdown, and multiple-choice questions (65). We employed a multistep process to collect the social network data, focusing first on developing social networks through name generators, followed by measures of demographic similarity, perceptual affinity (shared interest), and tie strength (66,67). A series of 6 name generators, questions that elicit a list of contacts the participant knows, were used to develop the social networks. A combination of exchange, contact, and intimacy-based name-generator questions were used to create a list of network members (68,69). The name generators used focused on eliciting individuals in a participant’s network who can provide various forms of social support, social capital, and information (70). A time recall of 1 year was used to increase respondents’ recall of contacts (71). Alter (the connections of the network) information was collected from a series of supplemental measures, including demographic similarity, the strength of tie measures, perceptual affinity, and connectedness, to determine alter–alter ties (69,71). Participants were able to complete network questions using the tablet on their own, or with the help of the interviewer. When collected during phone conversations, the software provided a script for the interviewer to follow by recalling alter names. The questions used were based on design recommendations by Valente. A copy of the social network questions is available from the authors.

### Data Analysis

Data from the questionnaire were first analyzed descriptively. Demographic data were organized by frequency. Instruments were scored according to their scoring rubrics. A 2-sample *t* test was used to examine similarities and differences between aging gay individuals and aging lesbian individuals across instruments. The analysis was conducted in SPSS 25 for Macintosh. Social network data were initially coded in Excel and imported into the UCINET 6.6 social network analysis software package. All instruments and demographic data were calculated for all participants and then for gay and lesbian participants individually. Pearson’s correlation was utilized to examine correlations between health status and the measures of LGBT and rural identity congruence. A Pearson’s correlation was used to see if an individual’s perceived lesbian/gay identity congruence and rural identity correlated with the composition of the networks. Finally, given the importance of self-identification and the impact of self-identification as a gay or lesbian individual in terms of their livelihoods, health, and network development, an analysis of the relationship between identity congruence and quality of life was conducted.

The frequency of variables was calculated using UCINET and SPSS. Categorical alter attributes (descriptive factors) and homophily were computed. The demographic similarity score considers gender, sexual orientation, occupation, and geographic location of individuals and social contacts in calculating a final score on a scale of 0 (not at all similar) to 1 (very similar). The measures were summed, averaged, and compared. Perceptual affinity was calculated using means for individuals and populations using 4 questions. A final score was calculated using a final score of 0 (not similar) to 1 (very similar). The measures were then summed, averaged, and compared. Tie strength was calculated using a 4-scale measure focused on tie utilization. The measures were then summed, averaged, and compared. To calculate groupings of ties, the effective size of the networks was calculated using UCINET 6.6 for Windows. The effective size measures the number of contacts an ego has, minus the average number of ties each contact has to other contacts. Effective size measures redundancy in an egocentric social network, or what “pots of information” an ego can access (Perry, Pescosolido, and Borgatti, 2018, p. 181).

## Results

### Self-Reported Questionnaire

#### Demographics

Twenty-five individuals participated in the study. Participants were almost equally distributed between gay men (*n* = 13; 52%) and lesbians (*n* = 12; 48%); all but one participant identified as non-Hispanic White. Although not planned, it is not surprising this occurred. This recruitment mirrors the demographics of the region (72). The average age of all participants was 60.32 years (50–79). The average age of gay men (61 years; range = 52–79) was slightly higher than that of lesbians (59 years; range = 51–72).

Most participants (80%) received at least a postsecondary education. Overall, gay men reported higher education and income. Fifty-two percent of participants were employed. Most participants (72%) did not live alone. Fifty-six percent reported having children. Sixty percent reported having a religious affiliation.

Participants lived in 3 states: Kentucky (*n* = 20), Tennessee (*n* = 2), and West Virginia (*n* = 3). Five married couples participated in the study: 2 married gay and 3 married lesbian couples. Data collection occurred 4 years after the legalization of same-sex marriage. A description of participant characteristics is found in Table 1.

**Table 1.** Participant Characteristics

Characteristic		*n* (%)
Sex	Male	13 (52%)
	Female	12 (48%)
Sexual orientation	Gay	13 (52%)
	Lesbian	12 (48%)
Race/ethnicity	White	24 (96%)
	Asian	1 (4%)
Religious affiliation	Yes	15 (60%)
	No	10 (40%)
Relationship status	Married	12 (48%)
	Divorced	3 (12%)
	Widowed	1 (4%)
	Separated	0 (0%)
	Never married	4 (16%)
	Member of unmarried couple	5 (20%)
Education	Grades 9–12	1 (4%)
	High school graduate	4 (16%)
	College 1–3 years	8 (32%)
	College graduate	5 (20%)
	Graduate school	7 (28%)
Income	Less than $10 000	1 (4%)
	$10 000 to less than $15 00	2 (8%)
	$15 000 to less than $25 000	3 (12%)
	$25 000 to less than $35 000	4 (16%)
	$35 000 to less than $50 000	2 (8%)
	$50 000 to less than $75 000	9 (36%)
	$75 000 to less than $100 000	0 (0%)
	$100 000 or more	3 (12%)
Employment	Employed	13 (52%)
	Unable to work	4 (16%)
	Unemployed	2 (8%)
	Retired	6 (24%)
Children	Yes	14 (56%)
	No	11 (44%)
County of birth	A nonrural (KY, WV, TN) county	3 (12%)
	The rural county of residence in KY, WV, TN	10 (40%)
	Another rural (KY, WV, TN) county	2 (8%)
	Nonrural county outside of KY, WV, TN	4 (16%)
	Rural county outside of KY, WV, TN	5 (20%)
	Another country	1 (4%)
Live alone	Yes	7 (28%)
	No	18 (72%)

*Notes*: KY = Kentucky; TN = Tennessee; WV = West Virginia. The average age for gay and lesbian participants was 61.38 (range 52–79) and 59.16 (range 51–72), respectively.

#### Health

Most participants reported good (52%) or very good (12%) health. Lesbians (75%) reported higher rates of good/very good health than gay men (53.9%). Participants reported on average 3.64 (*SD* = 2.29) comorbid conditions, with gay men reporting a slightly higher number (4.23 [*SD* = 2.24]) than lesbians (3 [*SD* = 2.26]). Lesbians reported higher levels of disability than gay men. However, none of the numerical differences in health measures were statistically significant.

#### Access to services

Over half (*n* = 13) of participants reported being unable to access needed health care services. Sixty-four percent reported that needed services were not available in their community. The remaining (36%) reported they could not afford to access the service.

#### Quality of life

Based on the Flanagan QOLS, gay men (score = 71.92) had a numerically higher quality of life than lesbians (score = 69.92). Total participant scores (score = 70.92) fall slightly above the mean score of 69. For this assessment, a score of 90 is reported to be a “healthy population” (60). No statistically significant differences were found between gay and lesbian respondents. Findings are summarized in Table 2.

**Table 2.** Health and Identity Measure Results

Measure	Entire Sample	Lesbian	Gay	Sig (2-tailed)
Health				
Very bad, *n* (%)	0 (0.0%)	0 (0.0%)	0 (0.0%)	.6061
Bad, *n* (%)	3 (12%)	0 (0.0%)	3 (23.1%)	
Moderate, *n* (%)	6 (24%)	3 (25%)	3 (23.1%)	
Good, *n* (%)	13 (52%)	9 (75%)	4 (30.7%)	
Very good, *n* (%)	3 (12%)	0 (0.0%)	3 (23.1%)	
Disability, mean (*SD*)^*^	4.24 (5.85)	4.83 (6.26)	3.69 (5.63)	.787
Comorbid conditions, mean (*SD*)^†^	3.64 (2.29)	3 (2.26)	4.23 (2.24)	.185
Social isolation, mean (*SD*)^‡^	48.17 (13.27)	50.76 (11.11)	45.76 (15.03)	.358
Flanagan Quality of Life Scale, mean (*SD*)^§^	70.88 (17.22)	69.92 (21.84)	71.92 (11.16)	.779
LGBTQ identity				
Nebraska Outness Scale, mean (*SD*)^∥^^,^^⊥^	73.84 (26.80)	67.17 (27.26)	80 (25.87)	.239
Nebraska Identity Scale Disclosure, mean (*SD*)^¶^	36.48 (14.38)	33.08 (15.15)	39.62 (13.46)	.265
Nebraska Identity Scale Concealment, mean (*SD*)^#^	13.16 (13.91)	16.08 (13.9)	10.46 (13.9)	.323
Identity stigma, mean (*SD*)^**^	5.68 (2.27)	5.67 (1.83)	5.69 (2.69)	.978
Identity appraisal, mean (*SD*)^††^	21.20 (3.84)	20.92 (2.81)	21.46 (4.7)	.731
Community identity				
Rural identity, mean (*SD*)^‡‡^	18.80 (6.10)	18.50 (6)	19.08 (6.41)	.819
Community acceptance and identity, mean (*SD*)^§§^	21.60 (9.36)	21 (7.95)	22.15 (10.79)	.765

*Notes*: LGBTQ = lesbian, gay, bisexual, transgender, queer; *SD* = standard deviation.

^*^Average of self-reported disability.

^†^Average of self-reported comorbid conditions. The greater the number, the more comorbid conditions reported.

^‡^Scale of 34.8 to 74.2, with a median of 50. The higher the score, the more self-reported social isolation.

^§^Median of 69 and a healthy average of 90 in the general U.S. population. The greater the score, the higher the quality of life.

^∥^Scale of 0 to 100, with higher score indicating greater outness.

^¶^Scale of 0 to 50, with higher score indicating greater identity disclosure.

^#^Scale of 0 to 50, with higher score indicating greater identity concealment.

^**^Scale of 6 to 24, with higher score indicating greater identity stigma.

^††^Scale of 6 to 24, with higher score indicating greater identity appraisal.

^‡‡^Scale of 0 to 30, with higher score indicating greater rural identity.

^§§^Scale of 0 to 36, with greater score indicating greater community identity.

^⊥^Composite score of disclosure and concealment scales.

#### Identity congruence

Identity congruence measures focused on an individual’s lesbian/gay outness and rural identities. Measures included subsections of the NHAS and a modified Appalachian Identity Scale (61). The findings are summarized in Table 2. Gay men reported numerically higher levels of outness (score = 80; lesbian = 67.17) and identity disclosure (score = 39.62; lesbian = 33.08) and lower levels of identity concealment (score = 10.46; lesbian = 16.08) than lesbian individuals, as scored on the NOS. Gay men reported a slightly greater connection to a rural identity (score = 19.08; lesbian = 18.5) and connection to their rural community (score = 22.15; lesbian = 21) than lesbian individuals.

The second measure of LGBTQ identity, which focused on identity stigma and identity appraisal, did not show the same disparity between gay and lesbian participants. Aging gay men were more likely to disclose their sexual identity than lesbians. No statistically significant differences were noted between gay and lesbian individuals regarding identity congruence.

#### Social networks

Although lesbian networks are more extensive than gay networks (9.83 contacts vs 8.84), gay men (.89) reported greater tie strength compared with lesbians (.78). Participants reported an average network size of 9.32 (*SD* = 4.33) individuals. Aging lesbian participants reported more geographically distant networks (50% of contacts in the same county, 37.3% contacts in nonadjoining counties or outside of the state) than gay men, who reported their networks to be primarily located in the same county (61.7%) or an adjacent county (8.8%) than in nonadjoining counties (29.4%). Both groups reported networks consisting primarily of heterosexual/straight individuals. Gay men reported more lesbian individuals in their networks (17.8%) than lesbian individuals did gay men (3.4%). Both groups reported networks primarily consisting of family and friends. Lesbian participants reported more family members in their networks than gay participants. Gay men reported higher perceptual affinity (.69), the perceived similarity of interest between individuals and their social contacts than aging lesbian participants (.62). The higher level of perceptual affinity among aging gay men’s networks compared with aging lesbian networks was statistically significant at *p* = .0078. Table 3 summarizes the network characteristics of participants.

**Table 3.** Network Characteristic and Measures

Measure	Entire Sample	Lesbian	Gay	Sig (2-tailed)
Network characteristic, mean (*SD*)				
Number of ties	9.32 (4.33)	9.83 (3.97)	8.84 (3.49)	0.59
Perceptual affinity^†^	0.68 (0.07)	0.62 (0.06)	0.69 (0.08)	.0078
Tie strength^†^	0.83 (0.06)	0.78 (0.06)	0.89 (0.05)	.0001*
Demographic similarity^†^	0.56 (0.03)	0.58 (0.03)	0.55 (0.03)	.0262
Residence, *n*_County_ (%)^‡^				
Same county	130 (54.9%)	59 (50.0%)	71 (61.7%)	
Adjoining county	25 (10.5%)	15 (12.8%)	10 (8.8%)	
Nonadjoining county in the same state	27 (11.6%)	13 (11.0%)	14 (12.0%)	
Other	51 (21.9%)	32 (26.3%)	19 (17.4%)	
Sexual identity, *n* (%)^‡^				
Gay	27 (11.6%)	4 (3.4%)	23 (20.0 %)	
Lesbian	22 (9.4%)	21 (17.8%)	1 (0.9%)	
Straight	159 (68.2%)	77 (65.3%)	82 (71.3%)	
Bisexual	3 (1.3%)	2 (1.7%)	1 (0.9%)	
Unsure	8 (6.2%)	0 (0.0%)	8 (7.0%)	
Relationship, *n* (%)^‡^				
Family	103 (44.2%)	63 (53.4%)	40 (34.8%)	
Friend	65 (28.8%)	29 (24.6%)	36 (31.3%)	
Neighbor	22 (9.7%)	10 (8.5%)	12 (11.1%)	
Coworker	10 (4.4%)	4 (3.4%)	6 (5.6%)	
Acquaintance	7 (3.0%)	5 (4.2%)	2 (1.9%)	
Romantic partner	16 (6.9%)	7 (5.9%)	9 (8.3%)	
Other	4 (1.8%)	0 (0.0%)	3 (2.8%)	
Effective size				
Effective size score (*SD*)	7.21 (4.27)	7.48 (3.97)	6.97 (4.97)	.77

*Notes*: *SD* = standard deviation.

^†^Scored .00 (least similar) to 1 (most similar).

^‡^Percent may not add to 100% due to participants opting out of responding. Proportions reported in the rows are the proportion of the ego’s networks made up of these characteristics.

*p ≤ .0001.

The aging lesbian networks showed significantly (*p* = .0262) greater demographic similarity (.58) than aging gay men’s networks (.55). The final measure calculated was tie strength, the attachment one feels to the social contact (73). Aging gay men (.89) reported statistically stronger (*p* = .0078) network ties than aging lesbian females (.78).

### Patterns of Social Networks

Demographic similarity was used to determine the homogeneity of the networks. Lesbian individuals reported a slightly higher homogeneity score of .58 (*S.D.* = .031) than gay individuals at .55 (*SD* = .032). The difference in the homogeneity of networks between gay and lesbian participants was statistically significant at *p* = .0262.

Effective network size, the measure of the number of social connections an individual has, minus the connection’s relationship with one another, was calculated to understand the structure of the networks better and overlap among the social contacts. As participants’ networks were shown to be nonhomogenous, a smaller effective size would indicate that participant contacts would be connected. Participants had an average network effective size of 7.12 (*SD* = 4.27) and an average network size of 9.32 (*SD* = 4.33). Participants’ contacts do not appear to be highly connected. Gay individuals reported a smaller effective size at 6.97 (*SD* = 4.97, average network size of 8.84) than lesbians at 7.48 (*SD* = 3.55, average network size 9.8), meaning their networks were more restricted in access, as their connections had more connections with one another. The difference in effective size of networks between gay and lesbian participants was not statistically significant. A summary of findings can be found in Table 3. The networks do not appear constrained, and participants’ networks do not appear to consist of overlapping network members; they often keep separate networks.

A Pearson correlation was calculated based on the measure of effective size, the NOS score, and a rural identity scale score. A summary of the findings can be found in Table 4. None of the measures were a significant predictor of the composition of the members of the networks.

**Table 4.** Effective Size, Rural Identity, and Outness Score Correlations

Variable		Effective Size	Rural Identity	Outness Final
Effective size	Pearson correlation	1	.228	.057
	Sig. (2-tailed)		.273	.787
	*N*	25	25	25
Rural identity	Pearson correlation	.228	1	.184
	Sig. (2-tailed)	.273		.379
	*N*	25	25	25
Outness final	Pearson correlation	.057	.184	1
	Sig. (2-tailed)	.787	.379	
	*N*	25	25	25

The principal aim of this study was to discover the influence of social networks on health, identity, and quality of life. Two findings emerged. First, no statistically significant association between network size and quality of life was found. Second, there was a statistically significant (*p* = .014) relationship between identity congruence and quality of life. When individuals had greater agreement between internal feelings of self and external behaviors (or identity congruence), they reported higher rates of quality of life and health. The findings are shown in Tables 5 and 6.

**Table 5.** Correlation Between Network Size and Quality of Life

Variable		Network Size	Quality of Life
Network size	Pearson correlation	1	.372
	Sig. (2-tailed)		.067
	*N*	25	25
Quality of life	Pearson correlation	.372	1
	Sig. (2-tailed)	.067	
	*N*	25	25

**Table 6.** Correlation Between Quality of Life and Identity Congruence

Variable		Quality of Life	LGBTQ Identity Congruence
Quality of life	Pearson correlation	1	.487^*^
	Sig. (2-tailed)		.014
	*N*	25	25
LGBTQ identity congruence	Pearson correlation	.487^*^	1
	Sig. (2-tailed)	.014	
	*N*	25	25

*Note*: LGBTQ = lesbian, gay, bisexual, transgender, queer.

^*^Significant at the .05 level (2-tailed).

## Discussion

Rural environments appear to affect the development and compositions of social networks in our sample, however, not in the direction suggested by previous social network studies and theories (69,74). Rather than supporting more homogenous networks, the development of their networks was based on who was available to them. Thus, other factors, such as the need for support in aging, override the influence of gay or lesbian identity (54).

Much of the literature on rural lesbian and gay individuals highlights the importance of urban environments for developing networks and accessing resources. However, that was not present in this study (75–77). Our study population did not rely on urban-based individuals for support and information, rather relying on those who were closest to them geographically. As rural residents, results suggest participants focused on developing networks close to them, even if that meant networks that were not as supportive or helpful as they desired. Participants developed networks even if the networks required the concealment of their sexual identity. Lesbians reported greater identity concealment than gay men. Rather, it appears they rely on one of their other intersectional identities to create the connection, even if it means they might be required to self-conceal their sexual identity (78).

Contrary to existing research, there was no relationship between the size of an individual’s social network and quality of life (79). However, there was a positive correlation between an individual’s identity congruence and quality of life. Individuals who reported greater identity congruence also had higher quality-of-life outcomes. As with other findings in this study, this may be due to the limited sample size and lack of sample power.

### Network Development

At the start of this study, we assumed that larger networks would result in increased access to information and resources and thus greater self-reported quality of life. Contrary to previous research findings regarding networks, denser social networks did not result in a reported higher quality of life (80–82). This study has shown that although networks may be large, the support provided may be lacking. Networks may be composed of those not accepting the participants’ identity or individuals chosen due to geographic closeness in their community. In this way, a network’s members may sabotage potential positive health outcomes and behaviors out of the necessity for the individual to conceal their identity. They may be unable to engage in behaviors viewed as out of the norm by their network members.

In reviewing the social connection name generators, many participants reportedly did not have individuals to discuss *LGBTQ issues* within their network. For our participants, networks were developed on need and geographic closeness, not necessarily on individuals who shared their interests or experiences. A larger network consisting primarily of individuals who hide their sexual identity could, in our opinion, lead to poorer health outcomes (83–85). It seems that network size was not associated with a higher quality of life due to individuals not feeling fully immersed with those around them. Once again, this finding must be presented against the small sample size.

Most participants indicated that urban areas were the primary locations they accessed health care and other community resources (86). In our study, rural gay and lesbian older adults did not report a high level of urban engagement in their networks, even though existing research indicates a reliance on urban networks because of the information LGBTQ urban network members can provide (87,88). One explanation for the lack of urban reliance may be that as individuals have aged, their networks have shrunk to the point that they now exclude urban participants due to challenges in maintaining relationships (89).

### Social Networks, Health, Identity, and Quality of Life

Findings supported the idea of a positive relationship between identity congruence and quality of life. Participants with greater identity congruence did report higher rates of quality of life. This finding confirms that individuals who limited the concealment of their sexual identity and externalized their internal feelings experienced a higher quality of life (16,90). We confirm this to be true in rural environments among gay and lesbian older persons. These differences in perspective may influence health outcomes and quality of life.

Aging gay and lesbian individuals have overcome many adverse societal events (eg, the medicalization of their identity, antigay and lesbian laws and practices, and fighting for equal rights) that have targeted their sexual identity throughout their lifetime (91). Even so, for those who developed a congruent identity, there appears to be opportunities for a higher quality of life. Individuals with higher identity congruence were more likely to create supportive networks. As a result, they may be more likely to have greater self-reported health. In this study, they are also better educated and have higher incomes than those with lower levels of identity congruence. Individuals with lower identity congruence may avoid seeking medical care or aging services out of fear of disclosing their sexual orientation (54). The lack of interaction with health professionals and service providers can result in higher rates of social isolation and decreased quality of life (92). They may not seek out new network members for fear of being ostracized as they have been their entire lives (93). These findings are similar to those that the fear of disclosing sexual identity negatively affects their health and well-being (57,61).

It does appear rural lesbian and gay individuals develop networks, in part, based on their lesbian and gay identity. In reviewing effective size, it seems they work to separate their LGBTQ and non-LGBT identifying networks. Whether this is a feature of rural environments or part of the lesbian and gay experience cannot be ascertained. Instead, it results from the complex social web of identification all individuals navigate (94). Individuals must contend with competing identities, such as lesbian or gay, race or ethnicity, gender expression, and notions of rural when meeting potential network members. They must make informed decisions on what identities are safe to disclose and which are not. Additionally, although rural environments seem to influence network development through access to potential network members, it is uncertain whether this results from their identification as lesbian or gay or a feature of rural environments (95). Rural environments have small populations; thus, the choice of network members is constrained.

### Future Directions and Research Recommendations

Future work should analyze participants’ self-reported social networks beyond network size and their feelings and descriptions of these networks. There are less-understood factors keeping individuals’ networks within rural environments, despite the challenges (eg, lack of health care, social support access, and the need for identity concealment).

Additionally, the rise of the internet and social media created new methods of developing and sustaining relationships (96). Although participants stated during the network data collection that they might engage in communication with urban individuals through social media or other electronic means, they did not necessarily consider them part of their networks (97). It appears because these social media contacts are not physically present, even though they provide support, they are not viewed as network members. As we continue to transition more of our lives online, revisiting this potential cohort effect and monitoring how it might change will be necessary (98).

Furthermore, targeted work focusing on experiences other than that of non-Hispanic White rural sexual minority individuals must be done (99). We must center the experiences of rural sexual minority populations of color. We must also expand our work to focus on sexual and gender populations that may face even greater challenges, including bisexual and transgender older persons.

Limited data exist focusing on older rural sexual minority populations (20,100). Even less exists relating to collecting social network data, much less that combines network, identity, and health data. Future work must directly engage with these populations through research codesigned with them (101). As noted by Flatt, Cicero, Kittle, and Brennan-Ing (47), there is a need to further engage in longitudinal data collection among LGBTQ populations. Codesigning longitudinal social network studies may provide one approach to further understanding health outcomes among these populations.

Finally, future work must recruit from a broader geographic region to provide a broader perspective on the experiences of lesbian and gay older adults. Work such as that conducted by Dakin, Williams, and MacNamara (102) shows one model of conducting region-wide work with LGBTQ populations.

### COVID-19 and Limitations

The data described in this article were collected before the COVID-19 pandemic. Recent data have indicated that older adults fared much better regarding isolation, loneliness, and social network support than initially thought (103,104). For our population, the ongoing pandemic most likely highlighted gaps in social support and access to resources. We know rural communities faced greater challenges than their urban counterparts throughout the pandemic due to preexisting inequities in access to health care, services, and broadband (105–107). Concurrently, the pandemic may have strengthened or supported the development of new social connections among neighbors and others who live in the community. Understanding this process would assist future interventions in building social networks for social support among community members.

### Limitations

In this research, it was impossible to fully describe the influence of age, sexual identity, and rural location/identity on networks; findings show a need for further research to refine this analysis. Independent of one another, age, sexual identity, and rural identity/location influence networks. Yet, the exact impact and how age, sexual identity, and rural identity/location mediate and moderate these effects remain unclear. With our sample, we could not determine if the limited network resulted from their lesbian and gay identity or just a feature of rural environments. Some of the null findings may be explained due to the small sample size and would differ with larger, more diverse samples. There is a need for similar work to engage larger, more diverse, samples with nonparametric testing. Although social network data collection generates a lot of data, there is a need for that data to be more precise.

Further research on a larger population and more specific measures and questions regarding identity congruence, networks, and identity is needed. There also were several challenges relating to the recruitment, identification, and engagement of participants. Given the rural nature of the sample, in these states, it was not possible to recruit through LGBTQ organizations in these communities. Snowball sampling proved problematic as individuals did not necessarily want to disclose others’ sexual identities, but also contributed to the homogenous nature of the sample. The sample demographics do not represent the rural aging lesbian and gay populations. Whereas the rural sample is like the areas overall racial/ethnic demographics, White, the gay, and lesbian community is much more diverse than the selected region.

Additionally, the limited geographic region examined is not representative of all rural areas of the United States. Even so, this study provides a critical glimpse at the social networks of an understudied population, rural aging lesbian and gay individuals.

## Conclusion

Through this work, we sought to expand the interface between gerontological research and social network analysis for marginalized populations (108,109). This work’s findings highlight the need to continue research into LGBTQ aging, specifically rural LGBTQ minority aging. Research is needed that examines not only the depth of the aging experience among LGBTQ individuals but also considers the role of the residential environment. The experience of rural aging LGBTQ minority populations has been overlooked in much of the gerontological scholarship. This is especially true regarding the experiences of rural older LGBTQ populations. Although our work focused solely on the experiences of rural older gay and lesbian individuals, it is critical to note that other groups, such as bisexual and transgender rural aging adults, may face even greater challenges. Moving forward, we must pay particular attention to the experience of these populations. By engaging rural aging populations in research, we can be better prepared to address their health challenges.

## References

[1] Bennett KJ , Probst JC, Vyavaharkar M, Glover SH. Lower rehospitalization rates among rural Medicare beneficiaries with diabetes. J Rural Health. Summer 2012;28(3):227–234. doi:10.1111/j.1748-0361.2011.00399.x 22757946

[2] Thiede BC , Brown DL, Sanders SR, Glasgow N, Kulcsar LJ. A demographic deficit? Local population aging and access to services in rural America, 1990–2010. Rural Sociol. 2017;82(1):44–74. doi:10.1111/ruso.12117 28757660 PMC5528146

[3] Rhubart DC , Monnat SM, Jensen L, Pendergrast C. The unique impacts of U.S. social and health policies on rural population health and aging. Public Policy Aging Rep. 2020;31(1):24–29. doi:10.1093/ppar/praa034 33462552 PMC7799382

[4] Afifi RA , Parker EA, Dino G, Hall DM, Ulin B. Reimagining rural: shifting paradigms about health and well-being in the rural United States. Annu Rev Public Health. 2022;43(1):135–154. doi:10.1146/annurev-publhealth-052020-123413 34910581 PMC11295601

[5] Miller CE , Vasan RS. The southern rural health and mortality penalty: a review of regional health inequities in the United States. Soc Sci Med. 2021;268:113443. doi:10.1016/j.socscimed.2020.113443 33137680 PMC7755690

[6] Cronin A , King A. Power, inequality and identification: exploring diversity and intersectionality amongst older LGB adults. Sociology. 2010;44(5):876–892. doi:10.1177/0038038510375738

[7] Westwood S. The myth of “older LGBT+” people: research shortcomings and policy/practice implications for health/care provision. J Aging Stud. 2020;55:100880. doi:10.1016/j.jaging.2020.100880 33272451

[8] National Academies of Sciences E, Medicine. Future Directions for the Demography of Aging: Proceedings of a Workshop. The National Academies Press; 2018:408. 29989766

[9] Guest MA. Sexual and gender identity in older age. In: Dodd S, ed. The Routledge International Handbook of Social Work and Sexualities. Routledge; 2021:195–204:chap 14.

[10] King S , Dabelko-Schoeny H. “Quite Frankly, I have doubts about remaining”: aging-in-place and health care access for rural midlife and older lesbian, gay, and bisexual individuals. J LGBT Health Res. 2009;5(1–2):10–21. doi:10.1080/15574090903392830

[11] Fredriksen-Goldsen KI , Kim HJ, Bryan AE, Shiu C, Emlet CA. The cascading effects of marginalization and pathways of resilience in attaining good health among LGBT older adults. Gerontologist. 2017;57(suppl 1):S72–S83. doi:10.1093/geront/gnw170 28087797 PMC5241752

[12] Fredriksen-Goldsen KI , Kim HJ. The science of conducting research with LGBT older adults—an introduction to aging with pride: National Health, Aging, and Sexuality/Gender Study (NHAS). Gerontologist. 2017;57(suppl 1):S1–S14. doi:10.1093/geront/gnw212 28087791 PMC5241759

[13] Orel NA , Coon DW. The challenges of change: how can we meet the care needs of the ever-evolving LGBT family? Generations. 2016;40(2):41–45.

[14] Croghan CF , Moone RP, Olson AM. Working with LGBT baby boomers and older adults: factors that signal a welcoming service environment. J Gerontol Soc Work. 2015;58(6):637–651. doi:10.1080/01634372.2015.1072759 26193473

[15] Fredriksen-Goldsen KI. Dismantling the silence: LGBTQ aging emerging from the margins. Gerontologist. 2017;57(1):121–128. doi:10.1093/geront/gnw159 28053011 PMC5241790

[16] Fredriksen-Goldsen KI , Bryan AE, Jen S, Goldsen J, Kim HJ, Muraco A. The unfolding of LGBT lives: key events associated with health and well-being in later life. Gerontologist. 2017;57(suppl 1):S15–S29. doi:10.1093/geront/gnw185 28087792 PMC5241757

[17] Muraco A , Fredriksen-Goldsen KI. Turning points in the lives of lesbian and gay adults age 50 and over. Adv Life Course Res. 2016;30:124–132. doi:10.1016/j.alcr.2016.06.002 28066158 PMC5215460

[18] Lyons A , Alba B, Waling A, et al. Recent versus lifetime experiences of discrimination and the mental and physical health of older lesbian women and gay men. Ageing Soc. 2021;41(5):1072–1093. doi:10.1017/s0144686x19001533

[19] National Academies of Sciences E, Medicine. Understanding the Well-being of LGBTQI + Populations. The National Academies Press; 2020:359. 33104312

[20] Kim HJ , Jen S, Fredriksen-Goldsen KI. Race/ethnicity and health-related quality of life among LGBT older adults. Gerontologist. 2017;57(suppl 1):S30–S39. doi:10.1093/geront/gnw172 28087793 PMC5241754

[21] Shiu C , Kim HJ, Fredriksen-Goldsen K. Health care engagement among LGBT older adults: the role of depression diagnosis and symptomatology. Gerontologist. 2017;57(suppl 1):S105–S114. doi:10.1093/geront/gnw186 28087800 PMC5241758

[22] Nelson CL , Andel R. Does sexual orientation relate to health and well-being? Analysis of adults 50+ years of age. Gerontologist. 2020;60(7):1282–1290. doi:10.1093/geront/gnz187 31909416

[23] Ramirez-Valles J , Dirkes J, Barrett HA. GayBy Boomers’ social support: exploring the connection between health and emotional and instrumental support in older gay men. J Gerontol Soc Work. 2014;57(2–4):218–234. doi:10.1080/01634372.2013.843225 24798546

[24] James W , Cossman JS. Long-term trends in Black and White mortality in the rural United States: evidence of a race-specific rural mortality penalty. J Rural Health. 2017;33(1):21–31. doi:10.1111/jrh.12181 27062224

[25] Marmot M , Allen JJ. Social determinants of health equity. Am J Public Health. 2014;104(suppl 4):S517–S519. doi:10.2105/AJPH.2014.302200 25100411 PMC4151898

[26] Fischer MM. Social exclusion and resilience: examining social network stratification among people in same-sex and different-sex relationships. Soc Forces. 2022;100(3):1284–1306. doi:10.1093/sf/soab019

[27] Walsh K , Scharf T, Keating N. Social exclusion of older persons: a scoping review and conceptual framework. Eur J Ageing. 2017;14(1):81–98. doi:10.1007/s10433-016-0398-8 28804395 PMC5550622

[28] Hillman J. The sexuality and sexual health of LGBT elders. Annu Rev Gerontol Geriatr. 2017;37:13–26. doi:10.1891/0198-8794.37.13

[29] Kittle Krystal R , Boerner K, Kim K, Fredriksen-Goldsen Karen I. The role of contextual factors in the health care utilization of aging LGBT adults. Gerontologist. 2022;63(4):741–750. doi:10.1093/geront/gnac137 PMC10167762 36048185

[30] Crenshaw KW. Mapping the margins: intersectionality, identity politics, and violence against women of color. Stanford Law Rev. 1989;43(6):1241–1299.

[31] Harris AC , Anderson KM, Bergen DJ. Intersecting identities, intersecting issues: exploring the needs of LGBTQ+ communities in Wisconsin. Hum Soc. 2020;44(2):198–220. doi:10.1177/0160597619864999

[32] Bromell L , Cagney KA. Companionship in the neighborhood context: older adults’ living arrangements and perceptions of social cohesion. Res Aging. 2014;36(2):228–243. doi:10.1177/0164027512475096 24860203 PMC4030643

[33] Academies TN. The social environment in rural America. In: Merchant J, Coussens C, Gilbert D, eds. Rebuilding the Unity of Health and the Environment in Rural America: Workshop Summary. The National Academies; 2006. 21850785

[34] Perry BL , Pescosolido BA, Borgatti SP. Egocentric Network Analysis: Foundations, Methods, and Models. Structural Analysis in the Social Sciences. Cambridge University Press; 2018.

[35] Smith KP , Christakis NA. Social networks and health. Annu Rev Sociol. 2008;34(1):405–429. doi:10.1146/annurev.soc.34.040507.134601

[36] Cohen S. Social relationships and health. Am Psychol. 2004;59(8):676–684. doi:10.1037/0003-066X.59.8.676 15554821

[37] Berkman LF , Glass T, Brissette I, Seeman TE. From social integration to health: Durkheim in the new millennium. Soc Sci Med. 2000;51(6):843–857. doi:10.1016/S0277-9536(00)00065-4 10972429

[38] Erosheva EA , Kim HJ, Emlet C, Fredriksen-Goldsen KI. Social networks of lesbian, gay, bisexual, and transgender older adults. Res Aging. 2016;38(1):98–123. doi:10.1177/0164027515581859 25882129 PMC4706743

[39] Xue X , Reed WR, Menclova A. Social capital and health: a meta-analysis. J Health Econ. 2020;72:102317. doi:10.1016/j.jhealeco.2020.102317 32497954

[40] Antonucci TC , Birditt KS, Sherman CW, Trinh S. Stability and change in the intergenerational family: a convoy approach. Ageing Soc. 2011;31(07):1084–1106. doi:10.1017/S0144686X1000098X 31798194 PMC6889888

[41] Kittle KR , Boerner K, Kim K, Fredriksen-Goldsen KI. Social resource variations among LGBT middle-aged and older adults: the intersections of sociodemographic characteristics. Gerontologist. 2022;62(9):1324–1335. doi:10.1093/geront/gnac021 35106592 PMC9579464

[42] Allen KR , Blieszner R, Roberto KA. Perspectives on extended family and fictive kin in the later years: strategies and meanings of kin reinterpretation. J Family Issues. 2011;32(9):1156–1177. doi:10.1177/0192513x11404335

[43] Rowan NL , Giunta N, Grudowski ES, Anderson KA. Aging well and gay in rural America: a case study. J Gerontol Soc Work. 2013;56(3):185–200. doi:10.1080/01634372.2012.754813 23548141

[44] Lin N. Social Capital: A Theory of Social Structure and Action. Cambridge University Press; 2002.

[45] Carstensen LL. The influence of a sense of time on human development. Science. 2006;312(5782):1913–1915. doi:10.1126/science.1127488 16809530 PMC2790864

[46] Kahn RL , Antonucci TC. Convoys over the life course: attachment, roles, and social support. In: Baltes PB, Brim O, eds. Life Span Development and Behavior. Academic Press; 1980:253–286.

[47] Flatt JD , Cicero EC, Kittle KR, Brennan-Ing M. Recommendations for advancing research with sexual and gender minority older adults. J Gerontol B Psychol Sci Soc Sci. 2022;77(1):1–9. doi:10.1093/geronb/gbab127 34216459

[48] Borders T. Advancing the field of rural health research: moving beyond simply documenting rural vs urban differences. J Rural Health. 2017;33(3–4):3–4. 28052514 10.1111/jrh.12227

[49] Moore KD. An ecological framework of place: situating environmental gerontology within a life course perspective. Int J Aging Hum Dev. 2014;79(3):183–209. doi:10.2190/AG.79.3.a 25622472

[50] Brofenbrenner U. Ecological models of human development. In: International Encyclopedia of Education. 2 ed. Elsevier; 1994.

[51] Krieger N. Proximal, distal, and the politics of causation: what’s level got to do with it? Am J Public Health. February 2008;98(2):221–230. doi:10.2105/AJPH.2007.111278 18172144 PMC2376874

[52] York Cornwell E , Goldman AW. Local ties in the social networks of older adults. J Gerontol B Psychol Sci Soc Sci. 2020;76(4):790–800. doi:10.1093/geronb/gbaa033 PMC7955986 32227105

[53] Parker T. Documentation RUCC Codes. USDA. https://www.ers.usda.gov/data-products/rural-urban-continuum-codes/documentation.aspx. Updated November 27, 2017. Accessed December 20, 2017.

[54] Kneale D , Henley J, Thomas J, French R. Inequalities in older LGBT people’s health and care needs in the United Kingdom: a systematic scoping review. Ageing Soc. 2021;41(3):493–515. doi:10.1017/S0144686X19001326 34531622 PMC8423450

[55] Davies M , Lewis NM, Moon G. Sexuality, space, gender, and health: renewing geographical approaches to well-being in lesbian, gay, bisexual, transgender, and queer populations. Geography Compass. 2018;12(5):e12369. doi:10.1111/gec3.12369

[56] Mulé NJ , McKenzie C, Khan M. Recognition and legitimisation of sexual orientation and gender identity (SOGI) at the UN: a critical systemic analysis. Br J Soc Work. 2016;46(8):2245–2262. doi:10.1093/bjsw/bcw139

[57] Prevention CfDCa. *Behavioral Risk Factor Surveillance System Questionnaire*. 2019:93. https://www.cdc.gov/brfss/questionnaires/pdf-ques/2017_BRFSS_Pub_Ques_508_tagged.pdf

[58] Suen LW , Lunn MR, Katuzny K, et al. What sexual and gender minority people want researchers to know about sexual orientation and gender identity questions: a qualitative study. Arch Sex Behav. 2020;49:2301–2318. doi:10.1007/s10508-020-01810-y 32875381 PMC7497435

[59] Subramanian SV , Huijts T, Avendano M. Self-reported health assessments in the 2002 World Health Survey: how do they correlate with education? Bull World Health Organ. 2010;88(2):131–138. doi:10.2471/BLT.09.067058 20428370 PMC2814481

[60] Burckhardt CS , Anderson LK. The quality of life scale (QOLS): reliability, validity, and utilization. Health Qual Life Outcomes. 2003;1(60):1–7. 14613562 10.1186/1477-7525-1-60 PMC269997

[61] Krok-Schoen JLP-W , Angela L, Dailey PM, Krieger Janice L. The conceptualization of self-identity among residents of Appalachian Ohio. Papers Commun. 2015;15(3):229–246.

[62] Mohr JJ , Kendra MS. The Lesbian, Gay, & Bisexual Identity Scale (LGBIS). 2012:2. *Measurement Instrument Database for the Social Science*.

[63] Meidlinger PC , Hope DA. Differentiating disclosure and concealment in measurement of outness for sexual minorities: the Nebraska Outness Scale. Psychol Sex Orientat Gend Divers. 2014;1(4):489–497. doi:10.1037/sgd0000080

[64] Eddens SK , Fagan MJ, Collins T. An interactive, mobile-based tool for personal social network data collection and visualization among a geographically isolated and socioeconomically disadvantaged population: early-stage feasibility study with qualitative user feedback. JMIR Res Protoc. 2017;6(6):e124. doi:10.2196/resprot.6927 28642217 PMC5500782

[65] Stadel M , Stulp G. Balancing bias and burden in personal network studies. Soc Netw. 2022;70:16–24. doi:10.1016/j.socnet.2021.10.007

[66] Brashears ME , Quintane E. The weakness of tie strength. Soc Netw. 2018;55:104–115. doi:10.1016/j.socnet.2018.05.010

[67] Bruyn AD , Lilien GL. A multi stage model of word-of-mouth influence through viral marketing. Int J Res Market. 2008;25(3):151–163.

[68] Chung KK , Hossain L, Davis J. Exploring sociocentric and egocentric approaches for social network analysis. Presented at: Second International Conference on Knowledge Management in Asia Pacific; 2005; Victoria University of Wellington New Zealand.

[69] Crossley N , Bellotti E, Edwards G, Everett MG, Koskinen J, Tranmer M. Social Network Analysis for Ego-nets: Social Network Analysis for Actor-centred Networks. Sage Publications; 2015.

[70] Marin A , Hampton KN. Simplifying the personal network name generator: alternatives to traditional multiple and single name generators. Field Methods. 2007;19(2):163–193. doi:10.1177/1525822x06298588

[71] Valente T. Social Networks and Health—Models, Methods, and Applications. Oxford University Press; 2010.

[72] Commission AR. The Appalachian region. 2017. https://www.arc.gov/appalachian_region/TheAppalachianRegion.asp

[73] Granovetter M. The strength of weak ties. Am J Sociol. 1973;78(6):1360–1380.

[74] Feld SL , McGail A. Egonets as systematically biased windows on society. Network Sci. 2020;8(3):399–417. doi:10.1017/nws.2020.5

[75] Butler SS. LGBT aging in the rural context. Annu Rev Gerontol Geriatr. 2017;37:127–142. doi:10.1891/0198-8794.37.127

[76] Portz JD , Retrum JH, Wright LA, et al. Assessing capacity for providing culturally competent services to LGBT older adults. J Gerontol Soc Work. 2014;57(2-4):305–321. doi:10.1080/01634372.2013.857378 24798180 PMC4416410

[77] Bradford JB , Putney JM, Shepard BL, et al. Healthy aging in community for older lesbians. LGBT Health. 2016;3(2):109–115. doi:10.1089/lgbt.2015.0019 27046541 PMC4994054

[78] Stein GL , Beckerman NL, Sherman PA. Lesbian and gay elders and long-term care: identifying the unique psychosocial perspectives and challenges. J Gerontol Soc Work. 2010;53(5):421–435. doi:10.1080/01634372.2010.496478 20603752

[79] Stokes JE , Moorman SM. Influence of the social network on married and unmarried older adults’ mental health. Gerontologist. 2018;58(6):1109–1113. doi:10.1093/geront/gnx151 28962022 PMC6215455

[80] Ajrouch KJ , Fuller HR, Akiyama H, Antonucci TC. Convoys of social relations in cross-national context. Gerontologist. 2018;58(3):488–499. doi:10.1093/geront/gnw204 28329836 PMC6281328

[81] Craveiro D. The role of personal social networks on health inequalities across European regions. Health Place. 2017;45:24–31. doi:10.1016/j.healthplace.2017.02.007 28258989

[82] Litwin H , Stoeckel KJ, Schwartz E. Social networks and mental health among older Europeans: are there age effects? Eur J Ageing. 2015;12(4):299–309. doi:10.1007/s10433-015-0347-y 28804362 PMC5549154

[83] Zhang J , Centola D. Social networks and health: new developments in diffusion, online and offline. Annu Rev Sociol. 2019;45(1):91–109. doi:10.1146/annurev-soc-073117-041421

[84] Ellwardt L , Wittek RPM, Hawkley LC, Cacioppo JT. Social network characteristics and their associations with stress in older adults: closure and balance in a population-based sample. J Gerontol B Psychol Sci Soc Sci. 2019;75(7):1573–1584. doi:10.1093/geronb/gbz035 PMC7424276 30888040

[85] Taylor HO , Taylor RJ, Nguyen AW, Chatters L. Social isolation, depression, and psychological distress among older adults. J Aging Health. 2018;30(2):229–246. doi:10.1177/0898264316673511 28553785 PMC5449253

[86] Wienke C , Hill GJ. Does place of residence matter? Rural-urban differences and the wellbeing of gay men and lesbians. J Homosex. 2013;60(9):1256–1279. doi:10.1080/00918369.2013.806166 23952922

[87] Jenkins Morales M , King MD, Hiler H, Coopwood MS, Wayland S. The greater St. Louis LGBT Health and Human Services Needs Assessment: an examination of the Silent and Baby Boom generations. J Homosex. 2014;61(1):103–128. doi:10.1080/00918369.2013.835239 24313255

[88] Lee MG , Quam JK. Comparing supports for LGBT aging in rural versus urban areas. J Gerontol Soc Work. 2013;56(2):112–126. doi:10.1080/01634372.2012.747580 23350566

[89] Carstensen LL , Fung HH, Charles ST. Socioemotional selectivity theory and the regulation of emotion in the second half of life. Motiv Emot. 2003;27(2):103–123.

[90] Fredriksen-Goldsen KI , Kim HJ, Shiu C, Goldsen J, Emlet CA. Successful aging among LGBT older adults: physical and mental health-related quality of life by age group. Gerontologist. 2015;55(1):154–168. doi:10.1093/geront/gnu081 25213483 PMC4542897

[91] Bränström R , van der Star A. More knowledge and research concerning the health of lesbian, gay, bisexual and transgender individuals is needed. Eur J Public Health. 2016;26(2):208–209. doi:10.1093/eurpub/ckv160 26342132

[92] Fakoya OA , McCorry NK, Donnelly M. Loneliness and social isolation interventions for older adults: a scoping review of reviews. BMC Public Health. 2020;20(1):1–14. doi:10.1186/s12889-020-8251-6 32054474 PMC7020371

[93] Yang J , Chu Y, Salmon MA. Predicting perceived isolation among midlife and older LGBT adults: the role of welcoming aging service providers. Gerontologist. 2018;58(5):904–912. doi:10.1093/geront/gnx092 28637322

[94] Orel NA. Families and support systems of LGBT elders. Annu Rev Gerontol Geriatr. 2017;37:89–109. doi:10.1891/0198-8794.37.89

[95] Williams KA , Dakin EK, Lipschutz A. LGBT+ older adults in rural south central Appalachia: perceptions of current and future formal service needs. J Gerontol Soc Work. 2022;65(2):217–238. doi:10.1080/01634372.2021.1953661 34266367

[96] Van Orden KA , Bower E, Lutz J, et al. Strategies to promote social connections among older adults during “social distancing” restrictions. Am J Geriatr Psychiatry. 2021;29(8):816–827. doi:10.1016/j.jagp.2020.05.004 32425473 PMC7233208

[97] Clark R , Moloney G. Facebook and older adults: fulfilling psychological needs? J Aging Stud. 2020;55:100897. doi:10.1016/j.jaging.2020.100897 33272457

[98] Szabo A , Allen J, Stephens C, Alpass F. Longitudinal analysis of the relationship between purposes of internet use and well-being among older adults. Gerontologist. 2019;59(1):58–68. doi:10.1093/geront/gny036 29688332

[99] Stone AL. The geography of research on LGBTQ life: why sociologists should study the South, rural queers, and ordinary cities. Sociol Compass. 2018;12(11):e12638. doi:10.1111/soc4.12638

[100] Boykin KO. Where rhetoric meets reality: the role of Black lesbian and gays in “queer politics.” In: Rimmerman C, Wald K, Wilcox C, eds. The Politics of Gay Rights. University of Chicago; 2000.

[101] Scott D. Stigma in place: Black gay men’s experiences of the rural South. Health Place. March 2021;68:102515. doi:10.1016/j.healthplace.2021.102515 33515909

[102] Dakin EK , Williams KA, MacNamara MA. Social support and social networks among LGBT older adults in rural southern Appalachia. J Gerontol Soc Work. 2020;63(8):768–789. doi:10.1080/01634372.2020.1774028 32558626

[103] Barnes TL , Ahuja M, MacLeod S, et al. Loneliness, social isolation, and all-cause mortality in a large sample of older adults. J Aging Health. 2022;34:883–892. doi:10.1177/08982643221074857 35234547 PMC9483694

[104] Ernst M , Niederer D, Werner AM, et al. Loneliness before and during the COVID-19 pandemic: a systematic review with meta-analysis. Am Psychol. 2022;77:660–677. doi:10.1037/amp0001005 35533109 PMC9768682

[105] Karim SA , Chen H-F. Deaths from COVID-19 in rural, micropolitan, and metropolitan areas: a county-level comparison. J Rural Health. 2021;37(1):124–132. doi:10.1111/jrh.12533 33155723

[106] Callaghan T , Lueck JA, Trujillo KL, Ferdinand AO. Rural and urban differences in COVID-19 prevention behaviors. J Rural Health. 2021;37(2):287–295. doi:10.1111/jrh.12556 33619836 PMC8013340

[107] Henning-Smith C. The unique impact of COVID-19 on older adults in rural areas. J Aging Soc Policy. 2020;32(4-5):396–402. doi:10.1080/08959420.2020.1770036 32475255

[108] Fiori KL , Consedine NS, Merz EM. Attachment, social network size, and patterns of social exchange in later life. Res Aging. 2011;33(4):465–493. doi:10.1177/0164027511401038

[109] Rowan NL , Beyer K. Exploring the health needs of aging LGBT adults in the cape fear region of North Carolina. J Gerontol Soc Work. 2017;60(6–7):569–586. doi:10.1080/01634372.2017.1336146 28641046

